# Taking the long view: the development of the Well-being of Future Generations (Wales) Act

**DOI:** 10.1186/s12961-020-0534-y

**Published:** 2020-03-27

**Authors:** Eleanor Messham, Sally Sheard

**Affiliations:** 1 Public Health Wales, Betsi Cadwaladr University Health Board, Preswylfa, Hendy Road, Mold, Flinshire, Wales CH7 5DY United Kingdom; 2 Department of Public Health and Policy, University of Liverpool, Whelan Building, Quadrangle, Liverpool, L69 3GB United Kingdom

**Keywords:** Policy-making, Wales, sustainable development, politics, health

## Abstract

**Background:**

The Well-being of Future Generations (Wales) Act, 2015 (WFG Act), aims to change the ways of working in Wales to be sustainable for the future. Goals within the WFG Act include ‘a healthier Wales’, ‘a more equal Wales’ and ‘a more prosperous Wales’. Reviewing key factors that led to Wales enacting this ground-breaking legislation is worthwhile for other legislatures around the world that may wish to create policy for future generations. We suggest that the drive of individual politicians, events at the time and recent history were the most critical issues in developing a more nuanced piece of legislation – the WFG Act.

**Methods:**

Ten interviews were conducted with those involved in the development of the WFG Act. Relevant documents were identified through systematic literature reviews and discussion with interviewees. Initial outcomes were tested against policy analysis frameworks, and Kingdon’s Multiple Stream Analysis was selected.

**Results:**

Key ‘policy entrepreneurs’ were found to be important, along with growing evidence of the problems of climate change and recommendations for sustainable development in Wales. The importance of context and capitalising on global and local events by policy actors was significant. A supportive environment, including the third sector, community groups, cross-party backing, committed civil servants and a change of ministers helped with momentum.

**Discussion:**

Policy-makers did not work directly with historians on developing the WFG Act. However, recent history was included with collaboration of the Wales Audit Office, who had evaluated the Welsh Government’s implementation of the duty to promote sustainable development. Sustainable, future-generation policies of other nations were also used to help shape the WFG Act. Kingdon’s Multiple Stream Analysis is a useful theory to interpret the timing and impact of this policy change.

**Conclusions:**

The problem of climate change, suitable policy solutions, political support, timing and, most importantly, policy entrepreneurs were all significant in the development of the WFG Act. Due to multiple factors, policy-makers in Wales have legislated for the long term, placing sustainability and the well-being of present and future generations at the heart of public services and government.

## Background

In 2015, the Welsh Government passed a ground-breaking piece of legislation [1] – the Well-being of Future Generations (Wales) Act 2015 (WFG Act), which represented a shift from the policies and direction of the Westminster-based, United Kingdom parliament. While the United Kingdom Government cut back on sustainability programmes [2], Wales forged ahead with a forward-thinking approach to protect the health and well-being of the population. Nikhil Seth, the Head of Sustainable Development for the United Nations, in a speech about the WFG Act, proclaimed “*What Wales is doing today, we hope the world will do tomorrow, action more than words is the hope for our future generations*” [3].

The WFG Act contains goals for ‘A healthier Wales’, ‘A more equal Wales’, ‘A Wales of cohesive communities’, ‘A globally responsible Wales’, ‘A Wales of vibrant culture and thriving Welsh Language’, ‘A prosperous Wales’ and ‘A resilient Wales’ [4]. This represented a shift by the Welsh Government towards a new vision for the Wales of the future.

Not only does the WFG Act require public bodies such as the National Health Service (NHS) and local councils to comply and work towards these goals, but it also includes the Welsh Government itself. The scope of the public bodies involved enables the WFG Act to have a diverse impact, both in the short and long term. ‘Future trends’ reports are included in the WFG Act and are to be used by public bodies to plan for the services they will provide for future generations [4]. Every year, ministers in the Welsh Government review milestones that have been set as part of the WFG Act. A total of 46 indicators were created, which are quantifiable and form part of the milestones, and enable monitoring of progress. Indicators include ‘gender pay difference’, ‘the ecological footprint of Wales’ and ‘healthy life expectancy at birth including the gap between the least and most deprived’ [5].

The WFG Act instituted a Future Generations Commissioner, whose role is to be a guardian for future generations [6]. The Commissioner is appointed by a cross-party panel of Welsh Government [7], and has over 30 staff. The office of the Commissioner is independent of, but funded by, the Welsh Government, with the Wales Audit Office responsible for auditing [6]. The Commissioner challenges public service boards and other public bodies who have responsibilities under the WFG Act. Reports are made and letters are sent to encourage good practice and ask for more progress in areas that require it. For example, a 2018 report by the Commissioner’s office found that cultural and environmental objectives were not being made, with a greater focus on economic and social well-being objectives [8].

According to political figures in Wales, the WFG Act was the first time a government had adopted the United Nation’s Sustainable Development Goals into law. It was particularly significant because it was developed in a time of global financial crisis, when the United Kingdom government was cutting back public services and benefits. The Welsh government was ground-breaking in legislating for equality, communities and the health of future generations rather than having a narrower focus on economics.

Many factors contribute to policy-making. A ‘one size fits all’ algorithm for government policy-making does not exist [9]. However, models can be applied to policy development to identify key factors, including key people, groups, events, timing and public involvement. In analysing the development of the WFG Act, we can see the impact of specific elements that were particularly important in influencing future policies with public health implications [10]. If critical parts of the process can be identified, they can inform how, and when, to lobby or intervene for future policy with health impacts [11].

This empirical qualitative research study aims to discover the important factors that enabled the WFG Act 2015 to be enacted. This study summarises the key events and actors that led to the Welsh government legislating for future generations.

### Conceptual framework: Kingdon’s multiple streams

Multiple theories for policy-making exist [12]. Theories range from the simple, such as Walt and Gilson’s policy triangle [10], to the more complex such as the Advocacy Coalition Framework of Sabatier [13]. Some theories include ideology, such as Weiss’ iterative model, where ideology, competes with interests and information [12]. Ideology within policy-making includes philosophy, principles, values and political orientation. Other theories, such as Historical Institutionalism, include how political structures inform policy-making [14].

Kingdon’s Multiple Streams Analysis acknowledges public opinion, timing and ‘policy entrepreneurs’ who can galvanise the process [15]. Kingdon uses three streams to represent the different elements that must come together to create a window of opportunity – ‘problem’, ‘policy’ and ‘politics’ [16]. After collecting the data, Kingdon’s theory was chosen as a suitable framework to analyse the multiple variables involved in developing the WFG Act. This is because multiple factors, timing and actors were important to the development of the WFG Act. Sabatier’s Advocacy Coalition Framework was considered less suitable as, although there were different groups involved with different priorities, these were not the groups that had the most impact on the development of the WFG Act. Walt and Gilson’s policy triangle and other simpler theories do not include a timing factor, which we judged important in the development of the WFG Act. Kingdon’s Multiple Streams Analysis includes timing in the window of opportunity and is therefore a suitable model to use [16].

This study seeks to enable other legislatures in creating effective policies to promote sustainable development and the well-being of future generations, through identifying key factors in the development of the WFG Act.

## Methods

### Study setting

Wales has a population of 3.1 million [17]. Welsh governance structures have changed dramatically since 1990. The Welsh Assembly was created after Wales received devolved powers from the United Kingdom, with the first elections held in 1999. The Government of Wales Act 2006 separated the executive from the legislature, with the formation of the Welsh Government as the executive body [18]. Following a referendum in 2011, the Assembly received primary law-making powers for specific purposes, free from interference from the United Kingdom government or parliament [18].

The first Government of Wales Act [19] legislated that sustainable development be promoted by the Wales Assembly. However, the Wales Audit Office [20] found that, although annual reports were being published, there was no significant difference to sustainable development in Wales from the schemes devised. The WFG Act, and the long-term aspirations it contains to support future generations, was therefore a significant shift from previous governance mechanisms.

### Study design

This is a qualitative study to explore the events and policy actors that led to the enactment of the WFG Act. It employs oral history interviews with key policy-makers and analysis of relevant documents to construct a detailed history of the development of the legislation, and then analyses the findings using the Multiple Streams Analysis developed by Kingdon.

### Document review

We conducted a systematic search for relevant literature in peer-reviewed journal articles and grey literature. Scopus and JSTOR databases were initially searched using keywords relating to ‘sustainable development’, ‘health’ and ‘policy-making’ to find relevant peer reviewed articles. This was followed by searches of the Welsh Government and Welsh Assembly websites. Multiple combination searches of terms, including ‘sustainable development’, ‘well-being’ and ‘future generations’, were used on these sites to find relevant information. Grey literature was located via several mechanisms. Relevant grey literature items were mentioned by interview participants, with the most relevant documents referenced repeatedly by multiple interviewees. Brief mention of the WFG Act was found in two peer-reviewed journals [21, 22], but no in-depth analysis or study of the formation of the Act was found in the literature.

A spreadsheet was used to record the literature and news articles found. They were divided into different themes depending on what information they contained. For example, a news article about a Welsh minister’s public consultation was put under the ‘political actors’ heading and reports on global emissions were grouped under a ‘climate change’ heading. The headings evolved as more literature was found.

### Data collection through interviews

Online research on the WFG Act was used to create an initial list of key actors to request an interview with. A snowball approach was then used, asking interviewees for suggestions on who would be worthwhile to invite to participate. A range of respondents from a wide group of different organisations was aimed for to include those directly and indirectly involved and to give varied perspectives on the process. Interviewees were contacted by email, and after initial acceptance, were formally recruited to participate.

Of the 25 people approached for an interview by email, 2 declined and 13 failed to reply to the initial and follow-up email requests. Those who declined or did not respond included 4 civil servants within Welsh Government, 5 current or former Welsh Assembly members, 3 staff of the Commissioner’s office, 2 academics in climate change, a chief executive of a Welsh non-governmental organisation (NGO) and a member of the Welsh Local Government Association. The 10 who were interviewed are of similar positions to those who were approached, but not interviewed. See Table 1 for the positions of participants when interviewed and most relevant former position.

**Table 1:** Interviewee details

Name	Current post (when interviewed)	Dates	Former post	Dates
Jane Davidson	‘Pro Vice-Chancellor responsible for External Engagement and Sustainability’ at the University of Wales, Trinity Saint David	2011 to present	Minister for ‘Environment, Sustainability, and Housing’	2007–2011
Peter Davies	Portfolio of roles including, Chair of the Wales Council for Voluntary Action	2015 to present	‘Sustainable Futures Commissioner’ for Welsh Government.	2011–2015
			(2005–2011: Commissioner for Wales on the United Kingdom Sustainable Development Commission)	
Jeff Cuthbert	Police and Crime Commissioner’ for Gwent	2016 to present	Minister for Communities and Tackling Poverty	2013–2016
			(2014, change to ministerial portfolios, WFG Act moved to Minister for Natural Resources)	
Clive Bates	Director of the company: ‘Counterfactual Consulting’	2012 to present	‘Director General, Sustainable Futures’ Welsh Government civil servant	2009–2012
Mike Palmer	‘Director of Implementation and Performance’ the Future Generations Commissioner’s office	2016–2017	Project Manager for the Auditor General, Wales Audit Office	2001–2016
Jo Charles	Associate director, Public Health Wales	2005 to present		
Matthew Quinn	‘Distinguished visiting fellow’, Cardiff University	2017 to present	‘Director of Environment and Sustainable Development’, Welsh Government civil servant	2007–2017
			(former post had various name changes over the 10 years)	
Catherine Pearce	‘Future Justice Director’, World Future Council	2011 to present		
Neville Rookes	‘Policy officer for Environment’, Welsh Local Government Association	2012 to present		
Anon.	Civil servant for Welsh Government	2006 to present	(Worked on sustainable development)	

Ten semi-structured interviews took place via telephone or skype. Participants were almost all based in south Wales – the hub for Welsh decision-making, as the National Assembly for Wales and the Welsh Government are located in Cardiff. Organisations working with politicians are therefore most often based within the region. The interviews took place between May and June 2017, 2 years after the WFG Act became law. The interview schedule included questions on views of the key elements and actors that helped develop the WFG Act, personal opinions on the legislation, and advice for other legislatures wishing to create similar legislation.

### Analytical approach

The interviews and documents were analysed using qualitative thematic analysis. Transcribing the interviews enabled coded themes from each to populate a spreadsheet; another spreadsheet with the same themes was created for the documents. The themes found were Wales, United Kingdom and World events, significant people, NGOs, public sector, public health, previous policies, and Welsh Government/Assembly. After this process, it became clear that Kingdon’s [15] Multiple Streams Analysis model would be applicable to the themes. The codes within the themes were divided into ‘problem’, ‘policy’, ‘politics’ and ‘policy entrepreneurs’ as Kingdon describes; see Table 2 for details.

**Table 2:** Themes summarised using Kingdon’s Multiple Streams Analysis

Problem	Policy	Politics	Policy Entrepreneurs
Climate change	United Nations Sustainable Development Goals	United Kingdom parliament reduces commitment to sustainability	Jane Davidson
Welsh Government failing to promote sustainable development	Wales Audit Office sustainable development recommendations	Welsh Government new powers, creating constitutional legislation	Peter Davies
Underperforming public services	Politicisation of well-being	Active NGO groups	Multiple Welsh Government Ministers
	Active and engaged civil servants keen for change	Public support for protecting future generations	Civil servants
	Climate change solutions		

## Results

**Fig. 1:**
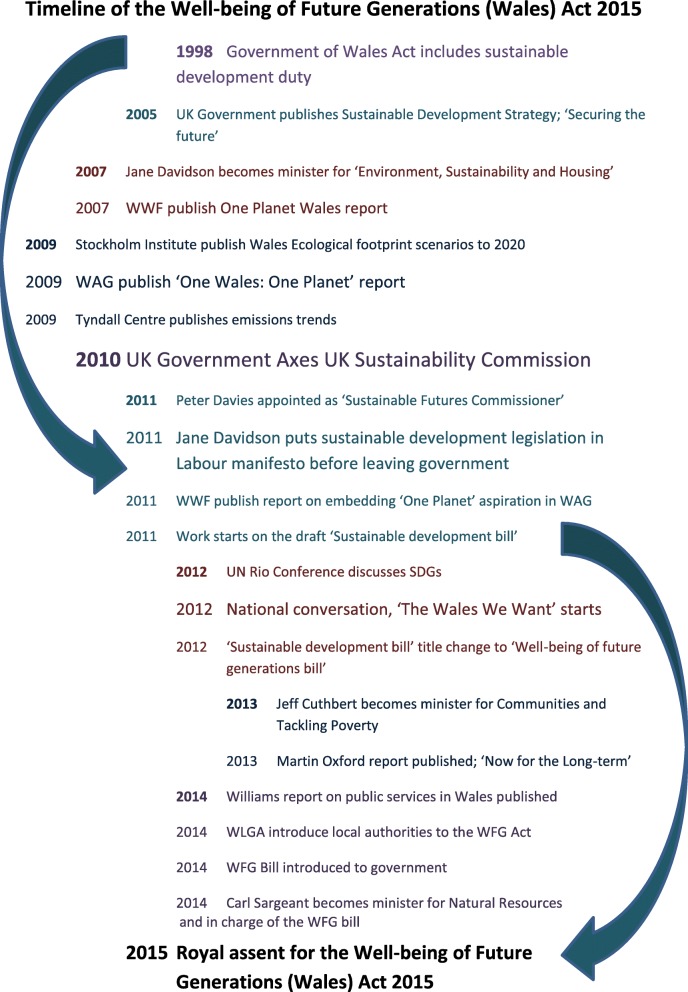
Timeline of events related to development of the WFG Act

A timeline displays the order of events that were significant to the development of the WFG Act (Fig. 1).

Kingdon’s multiple streams analysis [15], with the three streams, ‘problem, policy, politics’ and ‘policy entrepreneurs’ summarise the findings.

### Problem stream

The WFG Act did not respond to a specific problem, per se, as Kingdon [15] describes, as the Act was not created in response to a crisis or public dissatisfaction with the status quo. However, there had been calls to address climate change since 2007 [23]. The deficiencies of the duty to promote sustainable development in Welsh Government had also been documented [20]. Equally, the state of public services in Wales were found to be in need of improvement. The Williams [24] report found public services in Wales to be too crowded, complex and inadequately governed; the timing of the report’s publication meant it could be addressed in the WFG Act.

#### Climate change

The majority of interviewees note the importance of the climate change agenda and most notably the ‘One Planet Wales’ report of the World Wildlife Fund (WWF) [23]. The ecological footprint of Wales is the most striking part of the report that interviewees remembered.

“*The way we live in Wales has global impacts. If everyone consumed natural resources as we do, we would need three planets to support us.*” ( [23], p. 3).

Internationally, discussions on climate change were increasing with a United Nations climate change conference held in 2011 that committed to a climate change treaty [25]. Interviewees spoke of the imperative they felt to tackle climate change, including the former minister in Welsh Government for Environment, Sustainability and Housing, Jane Davidson,

“*… the Stockholm institute work, which was saying that with all our aspirations fully delivered, we would not decrease our ecological footprint. And since governments never deliver everything they plan to deliver, I was concerned that on my watch we’d end up with our ecological footprint being increased. But, the second element which is critically important, was that I set up the Climate Change Commission* [2007]*. Professor Kevin Anderson, who was then director of the Tyndall Centre at the University of Manchester, was giving us absolutely terrifying climate change data. And so the combination of those two things are what led to me wanting to propose the idea that we had to put sustainability at the heart of government.*”

#### Sustainable development duty

Seven of the ten interviewees noted the sustainable development duty of the 1998 Government of Wales Act [19]. Interviewees mentioned the need for further legislation because they felt the schemes and reports being produced were not producing substantive change. Peter Davies the former ‘Sustainable Futures Commissioner’ for Wales summarises,

“*We had then ten years or so of implementing that* [sustainable development] *duty… over that period there were evaluations undertaken by various bodies, by Cardiff University; commissioned by Welsh Government, Wales Audit Office, myself, when I was then commissioner, I prepared annual reports on the duty. All of those pieces of work pointed to the weakness in the application of the duty.*”

Various reports found the Welsh government did not fully promote sustainable development [20, 23, 26–29]. Due to the ‘problem’ of climate change and the ‘policy’ evidence of academic reports, this could be considered to straddle both the ‘problem’ and ‘policy’ streams of Kingdon’s model [15].

### Policy stream

Evidence and ideas for change, within the policy stream, came from the United Nations, Wales Audit Office, through the politicisation of well-being, and engaged and active civil servants within the Welsh Government.

#### United Nations

Seven of the interviewees discussed how the Sustainable Development Goals, developed at the 2010 Rio United Nations summit, had influenced the Welsh Government and the development of the WFG Act. Jeff Cuthbert, a former minister with responsibility for the WFG Act, is quoted in a report on the Act’s consultation process ( [26], p. 2):

“*Over the past two years there has been a global conversation, facilitated by the United Nations, with people the world over to seek their views on new sustainable development goals. In Wales too, we need to build a consensus around the goals which are the most important to all of us, including our children and grandchildren.*”

#### Wales Audit Office

As part of building consensus on the WFG Act, the Wales Audit Office (WAO) played a key role. The WAO advised on the WFG Act, in a break from its usual non-involvement position as an independent body. It was well-placed to contribute evidence from a 2010 report on Sustainable Development and Decision Making [20]. Many of the WAO’s recommendations can be seen in the WFG Act, for example, the need to legislate, to embed sustainable development within governance structures and to use sustainable development principles ( [20], p. 13–14). Clive Bates, a former civil servant observed,

“*The Wales Audit Office, they were an important actor. Getting to the heart of how money was not being used for the long-term. They could model the spending*.”

#### Civil servants

Other civil servants working for the three ministers who had responsibility for the WFG Act (John Griffiths, Jeff Cuthbert and Carl Sargeant) also played an active part. Almost all interviewees mentioned them as Peter Davies, former ‘Sustainable Futures Commissioner’ explains,

“*Over this period, we had some really good civil servants involved in it. … Who worked with stakeholders, and government structures to keep it moving along. That was a really important point*.”

The civil servants followed the ways of working that the WFG Act planned to introduce. The principles of involvement, collaboration and integration that they employed while developing the Act were proof that it could make a difference to government and beyond. This can be seen in the ‘involvement’ of members of the public in the consultation exercise, ‘collaboration’ of discussions with staff in the WAO and ‘integration’ by working alongside local councils who were early adopters of the principles of the WFG Act. Being able to see the Act put into practice helped persuade those sceptical of its potential impact to support it.

#### Politicisation of well-being

Civil servants inside Welsh Government contributed to significant developments in the language of the WFG Act. Initially entitled the Sustainable Development bill before the consultation process, it shifted, in 2012, to be known as the Well-being of Future Generations bill. The recognition of sustainability being more than just protecting the environment was taking root. Matthew Quinn, former Director of Environment and Sustainable Development for Welsh Government described the change,

“*The other thing that was happening was an interest in well-being, you had the new economics foundation, you had* [David] *Cameron being interested in happiness. An interest in well-being, which became a synonym for sustainable development. I think Clive Bates who was Director General, and my immediate boss for some of this period, he came from a campaigning background. He liked the well-being phrase, it appealed to him. He suggested we move from sustainable development which has baggage, and to the new buzz phrase of well-being. That’s why we ended up with the Well-being of Future Generations Act*.”

### Political stream

For the development of the WFG Act, the political stream had many elements. The United Kingdom parliament, Welsh Government, NGOs and the people of Wales were all active in developing the legislation.

#### United Kingdom Government

In 2010, the United Kingdom government closed the Sustainable Development Commission [2]. Commissioners from each of the four nations had been working together to create reports and ideas to further sustainable development within the United Kingdom [30].

Jane Davidson, the Minister responsible for sustainability at the time, explains,

“*The first event was in May 2010, the Sustainable Development Commission was having its tenth anniversary meeting, and we of course had just had the general election, with the Conservative- Liberal Democrat coalition, and they were expecting a minister to turn up to their special conference in Bristol. And not only did the Minister not turn up, but the official from DEFRA* [Department for the Environment, Food and Rural Affairs] *was not charged with any responsibility to communicate a message from the minister, and in a matter of weeks the Sustainable Development Commission had gone. So, in fact I wrote on the way back from Bristol on the train, I wrote some core elements about how one could have protected sustainable development.*”

Crises and policy changes are often linked together [15]. Although the closure of the commission might not be regarded as a crisis, it certainly helped focus the mind of some Welsh politicians to remain strongly supportive of sustainable development. The United Kingdom Sustainable Development Commission was an active group, producing reports and working together to further efforts on sustainable development across the four nations. Participation by representatives from Wales in the United Kingdom commission was deemed adequate by the Welsh Assembly at the time to fulfil obligations on promoting sustainable development. However, the closure of the United Kingdom commission coincided with a 2010 report by the Wales Audit Office that found the Welsh Government was not doing enough to make a difference on sustainable development. The Welsh Assembly at this point was required to produce annual reports on how the duty to promote sustainable development was being implemented internally. These reports were found to be ineffective in producing real change. These events together – the closure of the United Kingdom Sustainable Development Commission and the Wales Audit Office report – were confirmation that more needed to be done.

#### Welsh Government

Welsh politicians showed their support through the inclusion of legislation for sustainable development within the Wales Labour Party Manifesto [31]. Jane Davidson explains,

“*…but once it’s in the programme of government, I knew there were all those important elements that I thought were absolutely essential to take it forward, would have to be delivered. Because they were in the manifesto*.”

The wording within the Programme of Government reads, “*Legislate to make sustainable development the central organising principle of the Welsh Government and public bodies in Wales. Create an independent sustainable development body for Wales*” [27].

After the 2011 referendum, the Welsh Government was given new law-making powers, which became law in 2014. Two of the interviewees mentioned that the Welsh Government had only been in existence since 2000, and the development of the WFG Act, according to a former employee of the Wales Audit Office, was “*an example of the growing confidence of a fledgling institution*”.

Support for the WFG Act came from across this ‘fledgling’ Welsh Government. It was viewed as a constitutional piece of legislation as it aimed to change principles of governance, in particular the ways of working, within the government. Making decisions based on long-term results rather than short-term gains. All parties were involved and mostly supportive, as Mike Palmer, formerly of the Wales Audit Office explains “*For most of the period it was seen as a cross-party issue, at the actual vote it broke down, but that wasn’t to do with the act*”.

#### NGOs

The NGO Alliance, led by WWF, were openly critical of the WFG Act in 2015, causing a challenge for policy-makers. Seven of the 10 interviewees mentioned this group. WWF had commissioned a report that gave a roadmap to reducing the ecological footprint for Wales [32]. Jeff Cuthbert, one of the ministers with responsibility for the WFG Act described that “*We were lobbied hard by environmental groups and we had to resist that*”. A former civil servant explains the effects of the lobbying,

“*We nearly lost it at committee stage. That was due to the NGO group, led by WWF. They wanted to take it in an environmental way and wanted it to have bite on carbon change. They galvanised support against it*.”

The removal of environmental targets from the draft legislation was opposed by the environmental lobby. As mentioned above, the carbon targets were then included in the ‘Environment Bill’, which placated opposition parties and NGOs. A former civil servant responsible for the WFG Act noted that,

“*It was then decided to put action on climate change in the environment act, which it wasn’t initially intended to do. Carbon budgeting within the environment act was a promise made to the NGO lobby to enable the act to get through*.”

#### Public support

While the WFG Act was still in development, civil servants decided to change their own working culture and practices to reflect its key aims. “*We lived the values of the act in what we did*”, describes a former civil servant. To fulfil the ‘involvement’ way of working promise of the WFG Act, a national conversation was set up [33] – entitled ‘The Wales We Want’, it informed the development of the WFG Act. As the report states,

“*Through the conversation we asked people to discuss the Wales that they want to leave behind for their children and grandchildren, considering the challenges, aspirations and ways to solve long-term problems to create a Wales that they want by 2050. This process also helped to shape the six well-being goals that were contained in the Well-being of Future Generations (Wales) Bill when it was introduced in July 2014*.” ( [26], p. 2).

The consultative programme for the WFG Act differed from previous legislation, as an employee of the Welsh Local Government Association explains,

“*One of the things he* [Peter Davies] *did was not setting up a whole new consultative arrangement. He said, what groups meet normally, where are they? Let’s go and see them*.”

Almost 7000 people contributed to the discussions across Wales. Groups associated with health contributed, including Public Health Wales, the Royal National Institute of Blind People, Welsh NHS Confederation, Disability Wales and Wales substance misuse network [26].

### Policy entrepreneurs

Many individuals and organisations played a part in the development of the WFG Act. Policy entrepreneurs exploit ‘windows of opportunity’ in Kingdon’s Multiple Stream Analysis [15]. The vast majority of interviewees mentioned two names in particular – Jane Davidson and Peter Davies.

#### Jane Davidson

As Wales’ minister for Environment, Sustainability and Housing, 2007–2011, Jane Davidson was perfectly placed to have an impact on sustainable development. A civil servant describes her input,

“*The genesis for it was when Jane Davidson became Environment Minister, about 2008 I think. We currently had a Sustainable Development Scheme. She was very clear she wanted one that was more ambitious and more forward-looking, and that was a whole government scheme. That would reflect commitments from the whole of Government, rather than just being some environmental scheme.*”

They also commented, “*it was through her steel and determination that the act came into being*”.

A former Welsh Audit Office employee discusses her involvement,

“*Jane was the Minister responsible for this work* [sustainable development] *at this time. On the back of reports from various sources: academics, consultants, and the Wales audit report, she came to the conclusion that the only way to crack this was to legislate. She persuaded the government of the day that it needed to be included within the next manifesto.*”

Jane Davidson left politics in 2011, leaving what she describes as a “*bombshell*” for her colleagues to deal with.

#### Peter Davies

One of these colleagues was Peter Davies. Formerly the Welsh Commissioner on the United Kingdom Sustainable Development Commission, he was kept on as Wales’ Sustainable Futures Commissioner. A member of the NGO alliance discusses his input,

“*… Peter Davies, from where I was sitting he played an absolutely critical role. In building up the legislation, but also helping others identify their goals within the legislation. Peter is a very good networker. He let everyone know they had a role to play. He provided very strong leadership, but giving that leadership to others as well. … He made sure that everybody was heard.*”

Jane Davidson, former Minister, describes his contribution,

“*And his work has been absolutely extraordinary. ... Peter is the ultimate diplomat. Many, many, hours, days, months, years of work into trying to make sure that what happened in ‘the Wales We Want’ dialogue gave everybody, in every sector of life, an opportunity to contribute… A trusted expert, with a commitment to Wales and a commitment to sustainability. Who could work with environmental organisations, the social organisations, understood the whole charity sector, understood government, and facilitating. Absolute hero.*”

Two quotes from interviewees describe his involvement, “*Peter Davies was a tremendous critical friend of the government*” and “*If he hadn’t been there, it wouldn’t have moved as quickly as it did*”.

#### Moving around ministers’ portfolios

Peter Davies worked with three ministers (with different portfolios) while the WFG Act was being developed. The WFG Act moved from the Environment and Sustainable Development Minister, to the Minister for Communities and Tackling Poverty and, finally, to the Minister for Natural Resources, who got it to royal assent.

Despite sustainable development being prioritised across government following the creation of the Welsh Assembly, there was still resistance from Welsh decision-makers. Many in the Assembly needed persuading that the WFG Act would have the power to deliver outcomes and not be too bureaucratic. The multiple ministers responsible for the Act were able to use their different spheres of influence when they became knowledgeable and supportive of the WFG Act to persuade other Assembly members to back it. The constitutional nature of the Act, changing the ways of working, setting milestones etc., was not initially accepted by all in the Assembly. As a fairly new institution, passing such a wide-ranging, forward-looking policy had not been done before.

While Jeff Cuthbert was Minister for Communities and Tackling Poverty (2013–2016), he contributed to widening ‘The Wales We Want’ conversation. The former Sustainable Futures Commissioner, Peter Davies, describes,

“[he] *realised we needed a much bigger conversation around Wales and shaping the bill. Jeff initiated it and called for the conversation to happen*.”

Finally, the Minister for Natural Resources, Carl Sargeant, had the task of getting the bill through the Welsh Assembly. A civil servant at the time noted his contribution,

“*Carl Sargeant* [Minister for Natural Resources] *did a marvellous job of passing the legislation. … It went round the normal process. He was a plain speaking man who’d been chief whip, and he talked about it in normal terms. He championed it through.*”

The moves through different government departments and ministers is widely seen as a positive step, Peter Davies explains,

“*During the shifts between ministers it shifted out of the Environment ministers responsibility into being the Social Justice Minister’s responsibility. That was important, by accident rather than design. It brought in an audience that had a social justice agenda, rather than just an environmental agenda*.”

## Discussion

The study aimed to elucidate the factors that enabled the WFG Act to be enacted in Wales in 2015. The main factors were policy entrepreneurs, who capitalised on Kingdon’s [15] ‘window of opportunity’ when the problem, policy and politics streams came together. The main problem in need of a solution was climate change; the solution in the policy stream could be found in the United Nations Sustainable Development Goals and the Wales Audit Office recommendations for how the Welsh government could better promote sustainable development. The key political event was the United Kingdom government’s decision to close the United Kingdom Sustainable Development Commission in 2010. Two policy entrepreneurs who were instrumental to the development of the WFG Act were Jane Davidson and Peter Davies. The discussion will explore these factors.

Climate change and the potential harms for the planet motivated political actors in Wales to legislate. Research that showed that three planets would be needed if all countries used resources as Wales did was a significant finding, and hard to ignore [23]. The problem may have seemed stark but, without a viable solution, there could be no meaningful political action. The United Nations’ 2010 Sustainable Development Goals inspired politicians in Wales not only to legislate to reduce climate change, but also to include the public in deciding the direction that Wales should take. The new consultative process, led by Peter Davies, brought new people into the discussions and helped create broad support for the WFG Act. More locally, the Wales Audit Office 2010 report, which contained recommendations on how the Government could better promote sustainable development, established the necessary steps to change from the status quo [20]. The actions of politicians in the United Kingdom government to close the Sustainable Development Commission was a trigger for Jane Davidson, a minister in Wales at the time, to act to ensure sustainable development was not forgotten in Wales.

Kingdon’s model is a useful theory to apply to the development of the WFG Act, as it recognises that multiple elements must be present simultaneously for legislation to be enacted. This is certainly the case for the WFG Act, as there was no single actor or event that was instrumental in the development process. Rather, multiple factors in each of the streams of problem, policy and politics, with the policy entrepreneurs collectively keeping the ‘window of opportunity’ open, allowed the WFG Act to move from an idea to reality.

Kingdon [15] describes the three streams as separate; however, some of the factors of the WFG Act could be said to be part of two streams. An example of this is the action of the United Kingdom government in closing the Sustainable Development Commission – this was a political act and has been placed in the ‘politics’ stream; however, it could also be viewed as a ‘problem’, in that Wales had lost the ability to support sustainable development projects and learn from other nations within the United Kingdom. Likewise, the factor of climate change was certainly a ‘problem’, and has been defined as such in this study. This also had solutions and policy options thanks to research from universities and the third sector, meaning it could also be placed in the policy stream.

The change from the environmental focus to well-being and the movement through several minister’s portfolios enabled the WFG Act to have broader appeal. Initially entitled the Sustainable Development bill, before the consultation process, it was re-named as the Well-being of Future Generations bill in 2012. Along with the change of title there was also a wider appreciation of the more human side of sustainable development, with a focus on how prevention is better than cure in relation to health, how actions to protect the environment can simultaneously increase well-being, and that developing young people can lead to better employment prospects in the future. The environmental NGOs were not pleased when the WFG Act moved away from carbon reduction targets; however, they were placated by the agreement that they were included in other legislation. Nevertheless, the public and many stakeholders, through the ‘The Wales We Want’ consultation process, became advocates for legislating to promote well-being for future generations.

The bill’s move through different ministries also enabled the WFG Act to gain from the different perspectives and expertise of the ministers, each with their own views and agendas. For example, Carl Sargeant was initially reticent to support the WFG Act; however, when he could see that it was about people and communities, he became one of its strongest advocates. Moving from an environment minister's responsibility to Mr Sargeant's as minister for communities gave him the opportunity to champion the Act.

The WFG Act can be seen as a baton in a relay race, being passed initially from those who first ensured that sustainable development was to be promoted by the Welsh Government onto Jane Davidson. By including it within the Labour party manifesto, Davidson ensured that legislating for sustainable development would become part of the programme of government. Peter Davies then took on the baton and helped maintain momentum through leading on the development of the WFG Act alongside the different ministers. These policy entrepreneurs, as Kingdon [15] describes, were key factors in the development of the WFG Act.

The longevity of the WFG Act has yet to be tested. It is expected to take up to 10 years for the impact of the changes it has introduced to be seen. As with all legislation, it will warrant review as time passes. If the milestones that have been created as a result of it are not met, there could be reason to amend it. The Future Generations Commissioner's office has the responsibility to ensure the WFG Act is implemented and to measure its success [4].

There are several limitations in this study. Analysis was completed solely by the main author, which is more likely to be subjective than if this task were shared. Having more people analyse the data was not possible due to the constraints of the research, as it formed part of a Masters in Public Health project and also had associated time limitations. This also limited the number of people that could be interviewed. More perspectives could have added more information and depth to this research. The perspectives gained are more likely to be supportive of the WFG Act, as those who were approached but not interviewed are more likely to be sceptical or unsupportive. Although those known to be more sceptical or negative towards the WFG Act were invited to be interviewed, the majority of participants were in favour of the legislation. Whether the WFG Act does contribute to the well-being of future generations in a meaningful way is worth investigating. Further research once the legislation is fully implemented is recommended.

## Conclusion

The WFG Act development was successful due to several key factors, arguably the most important being the actors involved. Kingdon’s Multiple Stream Analysis theory [15] is appropriate for explaining the development of the WFG legislation. The problem stream contained the harms of climate change and a failure of the Welsh government to promote sustainable development. The policy stream included solutions from the politicisation of well-being to the United Nations Sustainable Development Goals and research recommendations for climate change. United Kingdom government action limiting developments in sustainability versus the widespread support within Welsh government, NGOs and the public for the well-being of future generations were all part of the politics stream. Policy entrepreneurs, most notably Jane Davidson and Peter Davies, advocated effectively for sustainable development in Wales and helped develop the WFG Act. The findings of this study are relevant to other policy-makers interested in the interplay between historical and current factors that enable countries’ politicians to take the long view and legislate for the well-being of future generations.

The WFG Act would be a significant achievement for an established government – it is quite surprising that such a ‘new’ legislature passed it. The Welsh Government had only been able to make laws for 4 years before the WFG Act was passed. Reviewing key factors has evidenced the importance of people in this ground-breaking legislation. Individuals at multiple levels, community groups, local councils, NGOs, civil servants, commissioners and ministers all contributed to the WFG Act becoming law. Without historical context and review these contributions may be lost, as they are not all chronicled and evidenced elsewhere. The motivation of key people made important contributions. Reports and evidence helped with this motivation, whilst the timing of local and world events also played their part. Understanding the process informs attempts to create new policy. Without an awareness of culture and individual’s stories and ambitions, policy development can struggle and at times fail. Studying how legislatures have crafted laws in the past, and especially how challenges are overcome, can lead to smoother enactments of subsequent legislation. Historical analysis, when used well to guide decision-makers, can enable learning from the past and inspiration for future generations.

## Data Availability

The datasets generated and analysed during the current study are not publicly available due to the nature of personal opinions expressed in the interviews. The full content of interviews have not been consented to be made public without checking with the individuals first. Interview transcripts are not publicly available to maintain anonymity of those who have requested it when consenting to be involved but are available from Eleanor Messham on reasonable request.

## Abbreviations

NGO: non-governmental organisation
NHS: National Health Service
WAO: Wales Audit Office
WFG Act: Well-being of Future Generations (Wales) Act 2015
WWF: World Wildlife Fund
